# Clinical correlates of emotion dysregulation in Down syndrome: a comparative study

**DOI:** 10.3389/fpsyg.2025.1632058

**Published:** 2025-09-24

**Authors:** Elisa Fucà, Valentina Maria Mongiovì, Stefano Vicari, Floriana Costanzo

**Affiliations:** ^1^Child and Adolescent Neuropsychiatry Unit, Bambino Gesù Children’s Hospital, IRCCS, Rome, Italy; ^2^Life Sciences and Public Health Department, Catholic University, Rome, Italy

**Keywords:** emotion dysregulation, lexical skills, trisomy 21, internalizing and externalizing symptoms, sleep disturbances, visual-motor integration

## Abstract

**Introduction:**

Emotion regulation is crucial for mental health and adaptive psychological functioning. Despite growing interest in emotion dysregulation (ED) in individuals with intellectual disability (ID), little is known about its association with neuropsychological and psychopathological profiles in children and adolescents with Down syndrome (DS), the most frequent genetic cause of ID. This study aimed to compare the neuropsychological profile, psychopathological symptoms, and sleep disturbances of children with DS with and without ED.

**Methods:**

Data were retrospectively collected from a database. The final sample included 214 participants (6–18 years): 69 with co-occurring ED and 145 controls without ED, with groups balanced for age and sex. Emotional/behavioral difficulties and sleep problems were assessed through parent-report questionnaires and clinical interviews. Language abilities and visual-motor integration skills were evaluated by standardized tests.

**Results:**

Children with co-occurring ED exhibited significantly worse visual-integration and motor coordination skills than children without co-occurring ED. Moreover, they displayed more psychopathological symptoms ascribable to both externalizing and internalizing problems. Finally, children with ED exhibited more sleep difficulties associated with insomnia and parasomnias.

**Conclusion:**

These results emphasize the need to consider ED in assessment and interventions, as it can significantly impact neuropsychological development and overall wellbeing.

## 1 Introduction

Emotion regulation refers to the process of adjusting various aspects of an emotional experience or response (Gross, 1998). Gross and Thompson (2007) describe five categories of effective emotion regulation strategies: situation selection, situation modification, attentional deployment, cognitive change, and response modulation. Effective emotion regulation helps keep the individual within a window of tolerance, allowing for optimal emotional functioning (Siegel, 1999; Greenberg, 2021). Therefore, emotion regulation is regarded as fundamental to mental health and adaptive psychological functioning. However, when emotion regulation fails or becomes ineffective, individuals may experience emotion dysregulation (ED). ED, indeed, occurs when there are deficits in adaptive regulation strategies or when ineffective responses are used, resulting in a mismatch between a person’s emotional expressions/experiences and the demands of the environment (Gross and Thompson, 2007). According to the distinction proposed by Cole et al. (2017) four types of dysregulated emotions associated with various forms of psychopathology can be identified. In the first case, emotions persist, and efforts to regulate them are unsuccessful (e.g., generalized anxiety and depression); in the second case, emotions disrupt appropriate behavior (e.g., disruptive behavior disorder). In the third case, emotions that are either expressed or felt are inappropriate for the context (e.g., posttraumatic stress disorder). Finally, emotions may shift either too rapidly or too gradually (e.g., bipolar disorder) (Cole et al., 2017). ED has been associated with poorer adaptive psychological and physical health outcomes, such as psychiatric symptoms, sleep disturbances and non-suicidal self-injury (Bradley et al., 2011; Wolff et al., 2019; Vanek et al., 2020).

Strategies of emotion regulation have been related with some neuropsychological correlates, such as language (Griffiths et al., 2021) working memory (Löytömäki et al., 2020) and cognitive abilities (Growney and English, 2023). Associations with disrupted sleep patterns have also been documented (Fisher et al., 2022). Since emotion regulation strategies often require complex cognitive skills, individuals with intellectual disability (ID) may be at higher risk of experiencing ED. For instance, in individuals with autism spectrum disorder and co-occurring ID, a mediating role of maladaptive emotion regulation strategies in relation to anxiety symptoms has been documented (Sáez-Suanes et al., 2020). A systematic review summarised the state of the art about ED in syndromic ID, reporting several studies that have suggested higher ED in individuals with ID [i.e., Down syndrome (DS), fragile X syndrome, tuberous sclerosis complex, Williams syndrome, Prader-Willi syndrome, and Angelman syndrome] in comparison with typically developing individuals (Shaffer et al., 2023). Some of these studies focused on DS, the most common genetic cause of ID. Challenging behaviors are common in youth with DS, with prevalence estimates reaching up to 100% (Mannion et al., 2024). Children and adolescents with DS may exhibit different kinds of behavioral issues depending on the developmental stage. Elevated levels of externalizing behaviors, such as impulsivity and temper tantrums, are frequently observed in early childhood and among school-aged children, whereas with maturation individuals with DS can experience higher rates of internalizing symptoms (Grieco et al., 2015). The systematic review by Shaffer et al. (2023) confirmed that individuals with DS exhibit ED, with inconsistent findings on potential sex differences and some evidence that behaviors related with ED tend to be stable among school-age children (Shaffer et al., 2023).

Despite the growing interest in investigating ED in individuals with ID, little is known about the neuropsychological and psychopathological profiles associated with ED in children and adolescents with DS. Thus, the current study had three aims:

- To investigate whether the presence of ED in children and adolescents with DS is associated with a distinct neuropsychological profile (e.g., cognitive, linguistic and visual-motor integration skills) compared to peers with DS without ED;
- To explore potential differences in the distribution of psychopathological symptoms between children with DS with or without ED;
- To examine whether children and adolescents with DS and ED exhibit more sleep problems than those with DS without co-occurring ED.

By addressing these dimensions, the findings may contribute to the development of targeted interventions and support strategies aimed at improving the overall wellbeing and quality of life for this vulnerable population.

## 2 Materials and methods

### 2.1 Procedure

This is a retrospective observational study with a cross-sectional analytic approach. Data were retrospectively collected from a file review of youth with DS referred for a clinical evaluation at the Child and Adolescent Neuropsychiatry Unit of the Bambino Gesù Children’s Hospital in Rome between July 2022 and June 2024. Participants underwent a clinical assessment consisting in a neuropsychiatric, neuropsychological and psychopathological/behavioral assessment. All parents signed a written informed consent for data use for research purposes and a privacy statement that ensures that data will be kept confidential. The study was conducted according to the guidelines of the Declaration of Helsinki and was approved by the local Ethics Committee (process number 2202_OPBG_2020; approved on 11 January 2021).

### 2.2 Participants

Selection criteria included a diagnosis of DS based on the analysis of the karyotype and the age ranging between 6 and 18 years. The diagnosis of DS was primarily based on external medical records. In a few cases, karyotype analysis was repeated at our institution. Among the 214 participants, four had mosaicism and three had translocation variants of trisomy 21. Exclusion criteria were age below 6 or above 18 years and language barriers preventing parents or caregivers from completing the questionnaires. The final sample consisted of 214 children with DS, of whom 69 had co-occurring ED (DS + ED group) and 145 were controls without ED (DS − ED group). The two groups were balanced for age and sex. Demographic features are summarized in Table 1.

**Table 1:** Demographic characteristics of the sample.

Characteristics	Total sample	DS − ED mean (SD)	DS + ED mean (SD)	*p*-values	Cohen’s *d*	95% CI of *d*
Age	11 (3.42)	11.67 (3.38)	11.66 (3.54)	0.988	0.004	[−0.283, 0.287]
Sex (M/F)	130/84	88/57	42/27	0.979	–	

### 2.3 Measures

#### 2.3.1 Emotion dysregulation

All participants were evaluated using the Child Behavior Checklist—CBCL (Achenbach and Rescorla, 2001), a 118-item scale completed by parents, which includes eight different syndrome scales, a Total Problem Score, and two broad-band scores, namely Internalizing Problems and Externalizing Problems. ED was evaluated through the CBCL dysregulation profile—DP, which is characterized by elevated scores across three specific syndrome scales: anxiety/depression, attention problems, and aggressive behavior (Hudziak et al., 2005); the CBCL-ED is calculated by adding the T-scores of these subscales. The total T-score is used as an index of ED, with higher values indicating greater dysregulation. Scores under 180 are considered within the normal range, values from 180 to 209 point to moderate dysregulation, while scores of 210 or above reflect severe ED. In line with previous literature (Frazier et al., 2015; Biederman et al., 2022; Conti et al., 2024), children were classified as belonging to the DS + ED group if they exhibited CBCL-DP score >180.

#### 2.3.2 Cognitive abilities

Cognitive abilities were tested by the Leiter-3 (Roid et al., 2013), a nonverbal assessment for individuals ages 3–70. The instrument explores the ability to reason by analogy, matching and perceptual reasoning, irrespective of language and formal schooling.

#### 2.3.3 Visual-motor integration

The Beery-Buktenica Developmental Test of Visual-Motor Integration (VMI) for individuals ages 3–18 (Beery and Buktenica, 1989) was used to assess visual-motor integration. The Beery VMI is comprised of drawings of geometric designs that increase in difficulty; participants are asked to observe the geometric designs and copy them with paper and pencil. Scoring criteria are based on the accuracy with which the designs were copied: higher scores indicate higher visual-motor ability. Raw scores are converted to standard scores according to the participant’s age. Beery VMI also includes two additional tasks: Visual Perception and Motor Coordination. Data from VMI were available for 175 participants, whereas data from the two additional tasks were available for 129 participants (DS − ED group *N* = 119; DS + ED group *N* = 56).

#### 2.3.4 Language abilities

The Phono-Vocabulary Test (Test Fono-lessicale—TFL) evaluates receptive and expressive vocabulary in children from 2 years and 5 months of age up to 6 years of age (Alfieri et al., 2022; Vicari, 2007). The subtest to evaluate receptive language consists of 45 tables with four images each: a target, a phonological distractor, a semantic distractor and a non-related distractor. The examiner pronounces a word illustrating one of the four pictures on the table and asks the participant to choose the picture that the word describes. The total score is determined by summing the correct responses provided by the child out of the 45 presented items. Additionally, it is possible to conduct a qualitative analysis of the performance by comparing how many times, out of 45, the child selected the phonological distractor instead of the target, how many times the semantic distractor was chosen, and how many times the distractor unrelated to the target was selected. The subtest to assess lexical production is made of the same pictures as the receptive subtest. The examiner points to a picture and asks the participant to name the target picture (1 point for each correct answer—45 items). For both subtests, it is possible to calculate raw scores and percentiles. In our study, we used the equivalent age values instead of the TFL percentiles. Equivalent age values are derived from raw scores and represent the chronological age at which the obtained score corresponds to the 50th percentile based on the instrument’s normative data. The TFL was administered to a subgroup of 158 participants (DS − ED group *N* = 114; DS + ED group *N* = 44), but only 126 participants completed the subtest to assess lexical production (DS − ED group *N* = 93; DS + ED group *N* = 33).

#### 2.3.5 Psychopathological symptoms

Schedule for Affective Disorders and Schizophrenia for School Aged Children Present and Lifetime Version DSM-5 (K-SADS). K-SADS is a semi-structured psychopathological interview that investigates the possible presence of psychopathological disorders according to DSM-5 (Kaufman, 2019). The K–SADS has a 3-point scale, where 1 = symptom is absent, 2 = symptom is present at a subclinical level, and 3 = symptom is severe and frequent enough to be at or above threshold. The K-SADS, as proposed in the instrument manual by Kaufman and collaborators, provides as a source of information not only the child/adolescent but also the parent(s). For some particular cases (i.e., ID), the parent is considered the main source of information with respect to the child. If general symptoms emerge in the screening interview, questions from the appropriate supplement are used to verify the diagnosis. We considered subthreshold symptoms to be subclinical psychopathology.

#### 2.3.6 Sleep disturbances

Sleep difficulties were assessed by means of Sleep Disturbance Scale for Children (SDSC) (Bruni et al., 1996), which explores the presence of sleep disturbances during the previous 6 months and contains 26 items with Likert scale values of 1–5. It is considered as “pathological” a T-score >70 and “suspect/borderline” a T-score between 61 and 70. The items are subdivided into six sleep disorder subscales: disorders in initiating and maintaining sleep (DIMS), sleep breathing disorders (SBD), disorders of arousal (DA), sleep–wake transition disorders (SWTD), disorders of excessive somnolence (DOES), and sleep hyperhidrosis (SHY).

### 2.4 Statistical analyses

Descriptive statistics (means, standard deviations, frequencies, and percentages) were calculated to characterize the demographic and clinical features of the sample. Between-group differences in categorical variables (e.g., sex distribution, prevalence of psychopathological symptoms from the K-SADS) were examined using chi-square tests. For continuous outcomes, independent-samples t tests were used to compare the two groups (DS + ED vs. DS − ED) when a single dependent variable was considered: nonverbal IQ (Leiter-3), receptive and expressive vocabulary (TFL), visual-motor integration, perception and motor coordination (Beery VMI) and age.

When multiple dependent variables were considered simultaneously, multivariate analyses of variance (MANOVA) were applied: SDSC subscales (sleep disturbance scores). Effect sizes were reported as partial eta squared (*η*_*p*_^2^) or Cohen’s *d*, which provides a standardized estimate of the proportion of variance explained by group differences. For key comparisons (e.g., SDSC sleep scores), 95% confidence intervals for *ηp*^2^ and *d* were also calculated to improve interpretability.

Assumptions of parametric analyses were considered satisfied given the relatively large sample size in each group (*n* > 30), according to the Central Limit Theorem, which states that the sampling distribution of the mean tends to normality as sample size increases, even if the underlying distribution is not perfectly normal (Lumley et al., 2002; Ghasemi and Zahediasl, 2012). Homogeneity of variances was checked with Levene’s test.

Missing data were not imputed, and analyses were conducted on available cases only. The sample size is reported for each analysis. All statistical tests were two-tailed, with significance set at *p* < 0.05. Analyses were conducted using SPSS version 22 (IBM Corp., Armonk, NY, USA).

## 3 Results

### 3.1 Group differences in neuropsychological profile

The investigation of potential differences in nonverbal cognitive abilities revealed that participants in the DS + ED group exhibited a mean IQ similar to that of participants in the DS − ED group (54.78 (6.25) and 55.53 (6.69), respectively, *p* = 0.431). Similarly, no differences between DS + ED (*N* = 44) and DS − ED (*N* = 114) groups emerged in linguistic abilities measured through the TFL, nor in comprehension [3.8 (1.33) and 4.09 (1.26), respectively, *p* = 0.249] or in production [4.11 (1.14) and 4.67 (2.19) respectively, *p* = 0.164; DS − ED group *N* = 93; DS + ED group *N* = 33].

The investigation of potential differences in visual-motor abilities revealed that participants in the DS + ED group (*N* = 56) performed significantly worse than children in the DS − ED group (*N* = 119) at the Beery VMI, in particular in Visual-Motor Integration [51.29 (8.64) and 54.6 (8.11) respectively, *p* = 0.015] and in Motor Coordination subtest [51.24 (6.84) and 54.37 (9.74) respectively, *p* = 0.04]. Conversely, no group differences emerged in the Visual Perception subtest [48.97 (7.74) and 50.69 (8.73), respectively, *p* = 0.296].

### 3.2 Group differences in the distribution of psychopathological symptoms

The analysis of the distribution of psychopathological symptoms as detected through the K-SADS revealed that participants belonging to the DS + ED group exhibited more psychopathological symptoms ascribable to both externalizing and internalizing problems. The results are summarised in Table 2.

**Table 2:** Distribution of psychopathological symptoms (%).

Domains	DS − ED (*N* = 145)	DS + ED (*N* = 69)
No symptoms	68%	27.5%
ADHD	13%	26.1%
Oppositional defiant disorder	3%	25%
Mood disorders	2.1%	5.8%
Anxiety disorder	4.8%	11.6%
Obsessive-compulsive disorder	1%	–
Tic disorder	3.4%	1%
Enuresis	1%	3%

### 3.3 Group differences in sleep disturbances

MANOVA detected significant group differences in SDSC scores, particularly at DIMS, DA, SWTD, and DOES scales. Table 3 summarises results.

**Table 3:** Differences between DS − ED and DS + ED groups in SDSC scores.

Subscales	DS − ED (*N* = 145) mean (SD)	DS + ED (*N* = 69) mean (SD)	*p*-values	*η*_*p*_^2^	95% CI of *η*_*p*_^2^
DIMS	55.46 (11.16)	63.81 (17.88)	<0.001*	0.076	[0.03, 0.14]
SBD	62.81 (16.68)	64.03 (17.14)	0.622	0.001	[0.000, 0.01]
DA	55.93 (14.37)	60.75 (17.15)	0.032*	0.021	[0.002, 0.06]
SWTD	56.39 (12.24)	63.71 (14.88)	<0.001*	0.064	[0.03, 0.12]
DOES	50.94 (10.43)	57 (13.72)	<0.001*	0.057	[0.02, 0.11]
SHY	49.92 (9.71)	52 (8.93)	0.125	0.011	[0.000, 0.05]

DIMS, disorders in initiating and maintaining sleep; SBD, sleep breathing disorders; DA, disorders of arousal; SWTD, sleep–wake transition disorders; DOES, disorders of excessive somnolence; SHY, sleep hyperhidrosis. *p < 0.05.

## 4 Discussion

The first aim of the current work was to investigate whether the presence of ED in children and adolescents with DS is associated with a distinct neuropsychological profile (e.g., cognitive, linguistic and visual-motor integration skills) compared to peers with DS without ED. We did not find group differences in nonverbal cognitive abilities. Although the design of the present study does not allow causal inferences about the relationship between ED and cognitive level, it is noteworthy that our results align with previous findings showing that the nonverbal reasoning does not predict success on a recognition of emotional facial expressions task in youth with DS, in contrast to what has been observed in typically developing children (Pochon et al., 2017).

We also found that children with DS and co-occurring ED did not differ in language skills from children with DS without ED. To our knowledge, this is the first study to examine language abilities in children with DS and ED using a comparative approach. Although research in typically developing populations suggests links between expressive vocabulary, working memory, and emotion recognition (Löytömäki et al., 2020), such associations may not generalize to DS due to their unique cognitive and neurological profile, which can influence how emotional regulation is acquired and expressed, independently of language proficiency. Previous research on adults with DS indicates that structural language level may be involved in mentalistic aspects of emotion understanding (Andrés-Roqueta et al., 2021), which is essential for basic emotion regulation, supporting the understanding of mental components such as attributing correct desires, beliefs, or intentions in stories. Our findings contribute to this line of research by examining associations specifically with ED rather than with basic regulatory skills. However, it should be noted that the present study assessed only lexical production and comprehension. Moreover, it used age-equivalent scores from a preschool-normed test to assess linguistic abilities in older participants, which may limit external validity. Nonetheless, this method was necessary to avoid floor effects and to meaningfully capture the participant’s abilities in this specific clinical sample (Edgin et al., 2010; Edgin, 2013; Esbensen et al., 2024). Future studies on the clinical features associated with ED in children and adolescents with DS could benefit from including more sensitive instruments and measures of other aspects of language and communication skills, such as verbal fluency or the comprehension of emotional prosody.

Children in the DS + ED group exhibited significantly worse visual-motor integration and motor coordination abilities, as measured by the Beery VMI test. This finding provides further support for the observation by Shaffer et al. (2023), who highlighted that cerebellar and hippocampal dysfunctions, coupled with impaired synaptic activity, lead to difficulties in motor coordination, learning, and memory in DS. In particular, the cerebellum is involved not only in motor control but also in emotional and cognitive regulation (Adamaszek et al., 2017; Stoodley and Schmahmann, 2018). It contributes to several key components of emotion processing, including physiological responses that underlie the subjective experience of emotions, emotional expressions that support social communication, and cognitive appraisal processes that evaluate whether stimuli elicit emotions and of what type (Baumann and Mattingley, 2022). This is in line with the universal hypothesis of cerebellar functioning, which proposes that the cerebellum optimizes accuracy, consistency, and appropriateness across both cognitive and affective domains, similar to its role in motor control (Baumann and Mattingley, 2022). Therefore, atypical cerebellar functioning in DS may represent a shared neural substrate linking motor impairments and increased ED.

At the same time, environmental factors may amplify these vulnerabilities. Children with poor motor skills often encounter repeated failures in everyday activities, reduced participation in peer play, and higher levels of parental stress, which can increase the risk of frustration and negative emotionality (Pimenta et al., 2023). In this perspective, Shaffer et al. (2023) suggested that, in DS population, motor coordination difficulties may heighten the risk of encountering frustration, which in turn may trigger the expression of ED. This interpretation is consistent with the findings from a study by Jahromi et al. (2008), who reported that children with DS exhibit limited repertoire for coping with frustration. Evidence from other developmental conditions reinforces this view: for instance, research on children with developmental coordination disorder has documented a connection between motor coordination problems and increased susceptibility to emotional and behavioral difficulties, including challenges in self-regulation (Green and Payne, 2018). An explanatory framework for this association can be traced in the Elaborated Environmental Stress Hypothesis (Cairney et al., 2013), which posits that poor motor skills increase the risk of internalizing problems through interactions with environmental stressors that act as intermediaries. Future studies should take into account the potential influence of environmental stressors on the relationship between motor coordination difficulties and ED in youth with DS. Given that individuals with DS are more prone to motor difficulties (Alesi et al., 2022), investigating these factors would enable a better understanding of the complex interactions between motor skills, mental health, and environmental influences, ultimately informing more effective interventions for children at risk. However, it is important to underline that, due to the cross-sectional design of the present study, no causal inferences can be drawn regarding the direction of the association between motor difficulties and ED.

The second objective of the present study was to explore potential differences in the distribution of psychopathological symptoms between children with DS with or without ED. Given that group assignment was based on the CBCL-DP, which already captures elevated internalizing and externalizing problems, the presence of more psychopathological symptoms in the DS + ED group might partly be expected. For this reason, we analysed the K-SADS data to offer a more objective and clinically detailed assessment, complementing the CBCL-DP classification. The structured clinical interview allowed us to specify diagnostic categories and quantify their prevalence in each group. Compared to the DS − ED group, children in the DS + ED group showed substantially higher prevalence of ADHD (26.1% vs. 13%), oppositional defiant disorder (25% vs. 3%), mood disorders (5.8% vs. 2.1%), and anxiety disorders (11.6% vs. 4.8%), along with a markedly lower proportion with no diagnosable symptoms (27.5% vs. 68%). These results indicate that, in children with DS, ED is associated with a broad spectrum of psychopathology, involving not only externalizing disorders but also internalizing conditions. This finding confirms and extends to the DS population previous evidence that ED reflects a restricted range of ineffective strategies for understanding or accepting one’s emotions, together with a limited repertoire for managing them (Gratz and Roemer, 2004), making it a factor that is strongly associated with psychopathological symptoms also in youth with DS. The results of the present study highlight the importance of early identification and tailored interventions to address the mental health needs of children with DS and co-occurring ED. Notably, since the existing literature on ED in individuals with ID mostly focused on externalizing behaviors (Te Brinke et al., 2021; Mcclure et al., 2009; Shaffer et al., 2023), our study emphasises the need for a comprehensive approach that also addresses ED and internalizing issues in this population, particularly in youth with DS. Further research is essential to clarify the underlying mechanisms and to develop more effective, individualised treatment strategies for children with DS and ED. Our findings, indeed, underscore the importance of systematically integrating ED monitoring into routine DS care. Parental training programs could play a crucial role by equipping caregivers with strategies to identify early signs of ED, promote adaptive responses in everyday contexts, and reduce escalation into more severe behavioral or emotional problems. A recent randomised feasibility pilot clinical trial explored the feasibility and acceptability of a parent training protocol for disruptive behaviors specifically designed for caregivers of children with DS, reporting decreased externalising behaviors, irritability and hyperactivity and improved behavioral regulation in executive functioning over time (Stone-Heaberlin et al., 2024). Promising intervention options may also arise from research evaluating the applicability and effectiveness of existing interventions targeting ED in populations with ID, such as Dialectical Behavior Therapy (DBT), as suggested by preliminary evidence (Florez and Bethay, 2017; McNair et al., 2017). Typical modifications to DBT involve simplifying concepts and language, repeating key content, incorporating visual supports, and providing additional assistance to help participants complete therapy material (McNair et al., 2017). Adapting evidence-based protocols such as DBT to the specific cognitive and social profile of individuals with DS may offer additional benefits, particularly if combined with psychoeducational components for families and teachers. Such approaches may foster a more comprehensive care model, in which ED is recognised and addressed as a central clinical feature, ultimately improving both emotional wellbeing and overall quality of life for children with DS and their families.

The last aim of this study was to investigate whether children and adolescents with DS and ED experience more sleep problems than those without co-occurring ED. Our results indicate that youth in the DS + ED group exhibited more sleep disturbances than those in the DS− ED group, particularly with respect to insomnia and parasomnias. The relationship between sleep and ED has been investigated by several studies, which have shown, for instance, that sleep deprivation reduces individuals’ ability to manage intense emotional reactions to highly negative stimuli (Yoo et al., 2007) and affects behavioral inhibition (Harrison and Horne, 1998), decision-making, and impulsivity (Killgore et al., 2006). These sleep-induced changes may work together to hinder the regulation of behaviors and emotions. Consistently, a study conducted in a sample of 476 college students found that participants self-reporting good sleep reported also significantly less ED than participants reporting more sleep difficulties (Fisher et al., 2022). Of note, the relationship between sleep and ED is thought to be bidirectional, as affective states can influence sleep patterns in various ways, such as through stress (Kahn et al., 2013). Our results confirm and extend to DS paediatric population previous findings in both typically developing children (Lustig et al., 2021) and clinical populations such as autism spectrum disorder (Northrup et al., 2024) and ADHD (Sanabra et al., 2022). However, given the distinct behavioral phenotype of DS, parallels with findings from other populations should be interpreted with caution, and are mentioned here only to highlight a broader pattern rather than to imply direct equivalence. As suggested by literature, the connections between sleep and ED are probably reciprocal, where sleep issues or lack of sleep worsen emotional and behavioral challenges, and ED interferes with sleep patterns. It should be noted that the results of the current study contrast with previous research indicating that children with DS and obstructive sleep apnoea display greater difficulties in emotional control than those without obstructive sleep apnoea (Joyce et al., 2020). The discussion on this inconsistency should take into account that in Joyce et al.’s study, obstructive sleep apnoea was specifically assessed using cardiorespiratory polygraphy in children aged from 36 months to just under 6 years, whereas in our study sleep breathing disorders were evaluated only through a screening questionnaire and the age range of participants differed. These differences in assessment procedures and participant characteristics are likely to account, at least in part, for the contrasting findings.

Our findings have important implication for the treatment of children and adolescents with DS and co-occurring ED, highlighting the need to address both sleep problems and emotional difficulties in an integrated manner. Treatments should consider the bidirectional relationship between sleep and emotions, as improvements in sleep may lead to a reduction in emotional and behavioral symptoms, and vice versa. Interventions aimed at improving sleep quality, such as sleep hygiene management or the introduction of relaxation techniques, could have positive effects on ED as well. At the same time, interventions targeting emotion management could help improve sleep patterns, thus reducing the negative impact of emotional disorders on sleep quality. A multifaceted therapeutic approach that integrates these aspects could be particularly beneficial for improving the overall wellbeing of children with DS and ED. At the same time, it should be acknowledged that the assessment of sleep relied solely on parent-report through the SDSC. Although this instrument is well validated, parental perceptions may be influenced by the child’s emotional and behavioral difficulties. This potential reporter bias suggests that our findings, while consistent with previous literature, should be interpreted with some caution.

The results of the present study should be interpreted in light of some limitations. First, as a comparative study, no causal inferences can be drawn regarding the relationship between ED and clinical characteristics. The study design does not allow for the determination of cause-and-effect relationships, and future research using experimental or longitudinal designs is needed to explore potential causal relationships. Second, the assessment of sleep disturbances relied on parent reports; future studies should incorporate additional measures, such as actigraphy, to provide more objective information on sleep quality. Third, not all participants completed the Beery-VMI and TFL subtests, resulting in unequal subgroup sizes across key measures. This may have introduced sampling bias and limits the generalizability of our findings. Finally, the cross-sectional design of the study limits the understanding of the temporal dynamins between ED and other clinical features. A deeper understanding of the relationship between ED and psychopathological, neuropsychological, and sleep profiles in DS would benefit from longitudinal studies with large samples.

Despite such limitations, the present study provides significant insights into the complexities of ED in children and adolescents with DS. Our results indicate that children with DS and co-occurring ED not only exhibit more pronounced psychopathological symptoms and sleep disturbances but also face greater difficulties in visual-motor integration compared to their peers without ED. These findings highlight the importance of considering ED when assessing and planning interventions for children with DS, as it may have significant implications for their neuropsychological functioning, behavioral profile, and overall wellbeing. Future studies could build on the framework proposed by Cole et al. (2017) to examine whether children with DS and co-occurring ED exhibit distinct patterns corresponding to the four subtypes of dysregulated emotions—persistent, disruptive, contextually inappropriate, or rapidly shifting. Understanding these different forms could offer deeper insight into the heterogeneity of ED clinical manifestations in this population and guide more tailored clinical assessments and interventions. Moreover, future research should continue to explore these associations, particularly through longitudinal studies, to better understand the long-term effects of ED on children with DS and inform more targeted interventions.

## Data Availability

The raw data supporting the conclusions of this article will be made available by the authors, without undue reservation.
